# Diagnostic performance of the lactate dehydrogenase-to-albumin ratio for early acute kidney injury in crush syndrome: a retrospective cohort study

**DOI:** 10.1186/s12873-026-01617-5

**Published:** 2026-05-23

**Authors:** Ayşenur Gür, Ramiz Yazıcı, Hüseyin Mutlu, Medine Akkan Öz, Yunus Yatmaz, Bensu Bulut, Murat Genç, Şükrü Yorulmaz, Hakan Güner, Mustafa Sırrı Kotanoğlu

**Affiliations:** 1 Department of Emergency Medicine, Etimesgut Şehit Sait Ertürk State Hospital, Ankara, Turkey; 2 Department of Emergency Medicine, University of Health Sciences, İstanbul, Turkey; 3 Kanuni Sultan Süleyman Training and Research Hospital, İstanbul, Turkey; 4 Department of Emergency Medicine, Aksaray University Medical School, Aksaray, Turkey; 5 Department of Emergency Medicine, Gülhane Training and Research Hospital, Ankara, Turkey; 6 Department of Emergency Medicine, Yenimahalle Training and Research Hospital, Ankara, Turkey; 7 Department of Emergency Medicine, Ankara Training and Research Hospital, Ankara, Turkey; 8 Ankara Training and Research Hospital, Ankara, Turkey; 9 Department of Emergency Medicine, Etlik City Hospital, Ankara, Turkey; 10 Department of Anesthesiology and Reanimation, Etlik City Hospital, Ankara, Turkey; 11 Ankara Provincial Health Directorate, Varlık Neighborhood, Halil Sezai Erkut Street No:5, Yenimahalle, Ankara, 06170 Turkey

**Keywords:** Crush syndrome, Acute kidney injury, Lactate dehydrogenase-to-albumin ratio, Prognostic biomarker, Emergency department

## Abstract

**Background:**

Crush syndrome is a life-threatening consequence of prolonged muscle compression, and acute kidney injury (AKI) is a major determinant of morbidity and mortality. Rapid AKI risk stratification at emergency department (ED) presentation remains challenging. We evaluated whether the lactate dehydrogenase-to-albumin ratio (LDAR) measured at ED admission could discriminate creatinine-defined AKI among patients with crush syndrome.

**Methods:**

We conducted a retrospective cohort study in the ED of a Level I trauma center from 1 January 2019 to 31 December 2025. Adults (≥ 18 years) meeting a predefined operational definition of crush syndrome, including traumatic compression injury, biochemical rhabdomyolysis, and compatible clinical or systemic features, were included. AKI was defined using serum creatinine-based Kidney Disease: Improving Global Outcomes criteria with a predefined approach for baseline creatinine determination. LDAR was calculated as admission lactate dehydrogenase divided by admission albumin. Discrimination was assessed using receiver operating characteristic (ROC) analysis with area under the curve (AUC) and 95% confidence intervals. Independent associations with AKI and RRT were examined using multivariable logistic regression.

**Results:**

Among 512 patients, 180 (35.2%) were classified as having creatinine-defined AKI; 374 (73.0%) were male. Hemodialysis was required in 78/180 (43.3%) patients with AKI, and in-hospital mortality was higher in patients with AKI (15.0% vs. 0.6%). Creatine kinase (CK) showed the highest discrimination for AKI (AUC 0.977, 95% CI 0.964–0.989), followed by LDAR (AUC 0.966, 95% CI 0.953–0.980). A Youden-derived LDAR cut-off of ≥ 23.59 yielded 86.7% sensitivity and 96.7% specificity. In addition, an LDAR cut-off of ≥ 22.68 predicted RRT requirement in patients with CS with 97.4% sensitivity and 77.6% specificity.

**Conclusions:**

LDAR measured at ED presentation showed high discrimination for AKI and RRT in crush syndrome. Although not a stand-alone treatment trigger, it may support early renal risk stratification when interpreted together with CK, renal function, electrolyte status, and clinical severity.

**Clinical trial number:**

Not applicable.

## Introduction

Crush syndrome (CS) is the most severe systemic consequence of prolonged skeletal muscle compression and can rapidly progress from a localized injury to life-threatening multi-organ dysfunction. Although it is most often discussed in the context of large-scale disasters—particularly earthquakes and structural collapse—it can also occur after high-energy trauma, such as road traffic collisions and industrial accidents [1, 2]. Reported incidence varies by population and case definition. In trauma cohorts, CS has been described in approximately 2–15% of patients, whereas considerably higher proportions have been reported in selected earthquake-related subgroups (e.g., hospitalized victims or those extricated after prolonged entrapment), reaching up to 45% in some series [2, 3].

The pathophysiological hallmark of CS is traumatic rhabdomyolysis. Myocyte disruption releases intracellular constituents—including myoglobin, potassium, phosphorus, uric acid, and other metabolites—into the circulation [4, 5]. Injured muscle compartments can also sequester large volumes of fluid, contributing to hypovolemia, renal hypoperfusion, and subsequent acute kidney injury (AKI) [4, 6]. Early metabolic complications, particularly hyperkalemia and early hypocalcemia, may further destabilize patients by precipitating arrhythmias and hemodynamic compromise [6]. Across published series, AKI in CS is consistently associated with higher resource utilization and worse outcomes: the need for renal replacement therapy and intensive care increases, and mortality rises when AKI develops, although absolute rates vary by setting and illness severity [7]. From an emergency department (ED) perspective, early risk assessment is clinically relevant—because initial resuscitation and electrolyte management are time-sensitive, and kidney injury may be evolving even before conventional markers fully reflect its trajectory.

A range of biomarkers has been proposed to support early AKI risk stratification, yet routine adoption remains limited by availability, cost, and variable performance across clinical contexts [8, 9]. Lactate dehydrogenase (LDH) is a widely available intracellular enzyme involved in glycolysis, present in many tissues [10]. Because LDH rises with cellular injury, elevated levels are common in tissue-damaging states, and higher LDH levels have been linked to worse clinical trajectories in conditions such as sepsis, acute pancreatitis, and renal dysfunction [10, 11]. Serum albumin, often viewed as a negative acute-phase marker, reflects inflammatory burden, physiologic reserve, and nutritional status and has been used as an indicator of illness severity in kidney injury [11]. Consistent with this, hypoalbuminemia has been associated with higher mortality in cohorts with sepsis and AKI [12, 13].

More recently, the LDH-to-albumin ratio (LDAR) has been examined as a simple composite index in several critical illnesses, with reports suggesting potential value in identifying patients at risk of adverse outcomes in sepsis, pulmonary embolism, and AKI [10, 14]. However, the role of LDAR in crush syndrome—particularly its ability to discriminate AKI risk at ED presentation—has not been well characterized. Therefore, our primary objective was to evaluate the diagnostic performance of admission LDAR for early identification of AKI in patients with CS after earthquake-related and non-earthquake trauma. Our secondary objective was to compare the discriminative performance of LDAR with routinely available clinical and laboratory parameters obtained at ED presentation.

## Methods

### Study design, setting, and ethics

We conducted a retrospective cohort study in the ED of a Level I trauma center from January 1, 2019, to March 31, 2025. The ED manages approximately 10,000 trauma-related visits per month. The study protocol was approved by the Bilkent City Hospital Ethics Committee (approval number: AEŞH-BADEK-2025-0612). Data extraction and analysis were performed only after ethics committee approval had been granted. The study was conducted in accordance with the Declaration of Helsinki. Because only routinely collected clinical data were analyzed and handled retrospectively, informed consent was waived.

### Participants and case identification

Adult patients (≥ 18 years) presenting after trauma—including road traffic collisions, occupational injuries, falls from height, and structural collapse—or injuries related to the 6 February 2023 earthquake were eligible for screening. Potential cases were identified using the institutional electronic health record and laboratory database.

Patients were included if they met a predefined operational definition of crush syndrome. Crush syndrome was defined as traumatic compression injury or prolonged entrapment/immobilization associated with biochemical evidence of rhabdomyolysis and at least one additional clinical, systemic, or local feature compatible with crush syndrome. Biochemical rhabdomyolysis was defined as CK > 1,000 U/L in the first blood sample obtained at ED presentation. Additional compatible features included dark/brown urine or documented myoglobinuria, electrolyte or acid–base abnormalities, hypovolemia or shock, clinically significant limb swelling or compartment syndrome, or the need for fasciotomy or amputation related to the crush injury. AKI was not used as an inclusion criterion because it was the primary outcome of the study.

A targeted chart review was conducted to confirm traumatic compression or prolonged immobilization and at least one compatible crush-related systemic or local feature. Patients with isolated CK elevation without documented compression injury or compatible crush syndrome features were excluded.

**Fig. 1:**
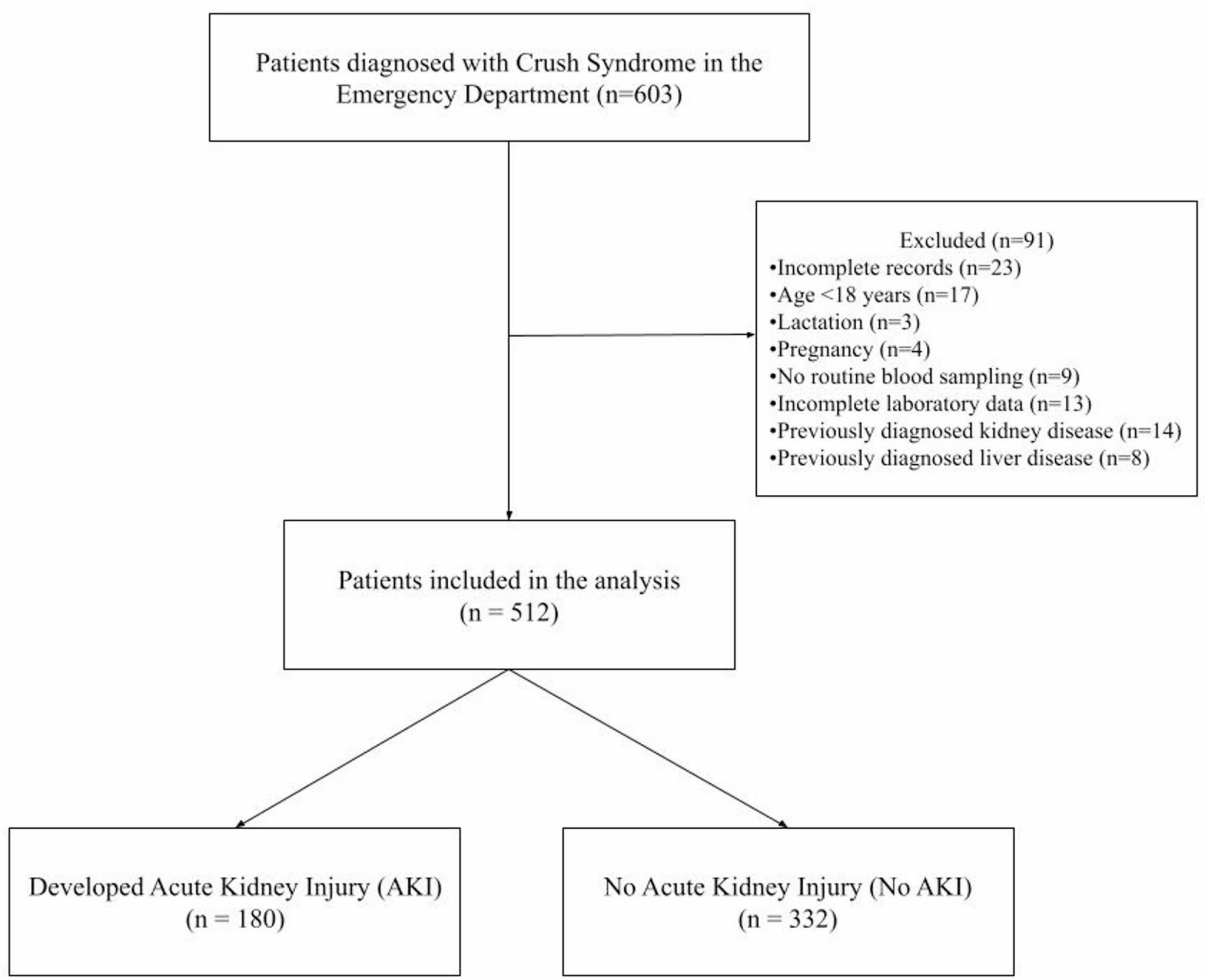
Patient flow

Patients were excluded if key admission laboratory results were unavailable, full records could not be retrieved, pregnancy or lactation was documented, or pre-existing chronic kidney or liver failure was documented. The patient selection process is summarized in Fig. 1.

### Data collection and variables

Data were extracted from the hospital’s electronic medical record system using a standardized data collection form. Key eligibility, exposure, and outcome variables were reviewed against the source records, including crush syndrome eligibility criteria, AKI classification, RRT requirement, mortality, LDH, albumin, CK, creatinine, and major clinical outcomes. Discrepancies or unclear entries were resolved by consensus with a senior investigator. Variables were grouped as follows:

Demographics and comorbidities: age, sex, and documented chronic comorbidities (including diabetes mellitus, hypertension, and coronary artery disease).

Etiology and injury characteristics: cases were categorized as earthquake-related or non-earthquake-related trauma. The primary anatomical region affected was categorized as upper extremity, lower extremity, or abdomen. Injury severity was quantified using the Injury Severity Score (ISS), calculated from documented injuries using standard definitions.

Resuscitation-related variables: red blood cell (RBC) transfusion and vasopressor requirement were recorded when present in the ED/hospital record.

Admission laboratory data: For discrimination analyses, we used the initial laboratory results obtained at ED presentation. Variables included complete blood count (white blood cell count, neutrophil count, lymphocyte count, hemoglobin, platelet count), serum biochemistry (creatinine, urea, alanine aminotransferase [ALT], aspartate aminotransferase [AST], lactate dehydrogenase [LDH], CK, albumin, sodium, potassium), and blood gas parameters (pH, partial pressure of carbon dioxide [PCO₂], bicarbonate [HCO₃⁻], lactate, and base deficit). Units were reported by the institutional laboratory. The baseline creatinine level was defined as the most recent stable pre-injury serum creatinine value recorded in electronic health records within the previous 12 months.

Clinical course and outcomes: length of hospital stay, intensive care unit (ICU) admission and ICU length of stay, need for hemodialysis, surgical interventions (fasciotomy and amputation), and in-hospital mortality.

Clinical management: management was not standardized for this study. Routine ED care commonly included intravenous fluid resuscitation and, when deemed appropriate by the treating clinician, urine alkalinization; mannitol was used selectively at the clinician’s discretion. Decisions regarding fasciotomy and amputation were made by the attending surgical teams. Hemodialysis initiation was determined after nephrology consultation. Detailed treatment variables, including prehospital fluid volume, exact in-hospital fluid rate and cumulative volume, timing and duration of urine alkalinization, mannitol dose and timing, duration of compression or entrapment, and duration of pre-/intraoperative hypotension, were not consistently documented in the retrospective records. Therefore, these variables could not be reliably included as quantitative covariates in the primary analysis.

### Exposure of interest: LDH-to-albumin ratio

The LDH-to-albumin ratio (LDAR) was calculated by dividing the admission LDH value (U/L) by the admission albumin value (g/L), using measurements obtained at ED presentation.

### Outcome definition

The primary outcome was AKI, defined according to the Kidney Disease: Improving Global Outcomes (KDIGO) serum creatinine criteria as an increase in serum creatinine of ≥ 0.3 mg/dL within 48 h or an increase in serum creatinine to ≥ 1.5 times baseline, known or presumed to have occurred within the previous 7 days. Secondary clinical outcomes were renal replacement therapy (RRT) requirement, ICU admission, and in-hospital mortality. RRT requirement was defined as the need for intermittent hemodialysis and/or continuous renal replacement therapy during hospitalization. RRT was initiated after nephrology consultation according to standard clinical indications, including refractory hyperkalemia, severe metabolic acidosis, volume overload, uremic complications, or persistent/worsening kidney dysfunction despite conservative management.

Baseline serum creatinine was determined using a hierarchical approach. When available, the most recent stable pre-injury serum creatinine value recorded in the electronic health records within the preceding 12 months was accepted as the baseline value.

Urine output criteria were not used in the primary AKI definition because hourly urine output was inconsistently and unreliably documented in the retrospective emergency department records. This approach was chosen to improve reproducibility and to minimize outcome misclassification related to incomplete urine output documentation in the acute care setting. Patients were classified into AKI and non-AKI groups, and baseline characteristics, admission parameters, trauma-related variables, and in-hospital outcomes were compared between groups. Because LDAR was calculated from the first blood sample obtained at ED presentation, and the exact timing of AKI onset could not be reliably determined in all patients, the primary analysis was interpreted as an assessment of admission-based discrimination/early identification of creatinine-defined AKI rather than prediction of strictly new-onset AKI after admission.

### Statistical analysis

Statistical analyses were performed using IBM SPSS Statistics (version 25). Distributional properties of continuous variables were assessed using the Kolmogorov–Smirnov test and visual inspection of histograms. Continuous variables are presented as median (interquartile range, IQR), and categorical variables as number (%). Between-group comparisons were performed using the Mann–Whitney U test for continuous variables and the χ² test for categorical variables; Fisher’s exact test was used when expected cell counts were low. All tests were two-sided, and p-values < 0.05 were considered statistically significant.

Discrimination for AKI was evaluated using receiver operating characteristic (ROC) curve analysis of admission clinical and laboratory measures. Areas under the curve (AUCs) are reported with 95% confidence intervals. Optimal cut-offs were selected by maximizing the Youden index, and sensitivity, specificity, positive predictive value (PPV), and negative predictive value (NPV) were calculated for the selected thresholds. Only variables available at emergency department presentation or before AKI classification were considered candidate predictors in ROC analyses and logistic regression models. Post-admission clinical course variables, including length of hospitalization, ICU length of stay, requirement for hemodialysis, and mortality, were excluded from predictive analyses because they occur after admission and may introduce reverse causality. ROC analysis was additionally performed to assess the discriminatory performance of admission LDAR for RRT requirement. ICU admission and mortality were not modeled as predictive outcomes and were reported descriptively.

Logistic regression was used to examine factors independently associated with AKI. Variables were assessed in univariable models and entered into the multivariable model if statistically significant (*p* < 0.05) or considered clinically important. The multivariable model was constructed using the backward stepwise likelihood ratio (LR) method. Model fit for the AKI and hemodialysis prediction models was assessed using the Hosmer–Lemeshow goodness-of-fit test. Serum creatinine was excluded from multivariable modeling to minimize incorporation bias, as it directly contributes to the KDIGO AKI definition. Adjusted associations are reported as odds ratios (ORs) with 95% confidence intervals (CIs). Multicollinearity was assessed using variance inflation factors (VIFs). Analyses were performed using complete cases.

Because this was a retrospective cohort study, no formal a priori sample size calculation was performed before data collection. All consecutive eligible patients during the study period were included. After reviewer recommendation, sample size adequacy for the diagnostic accuracy analysis was assessed using the Buderer method. This method estimates the required sample size according to the expected sensitivity and specificity, disease prevalence, confidence level, and desired absolute precision. Using a two-sided 95% confidence level, an AKI prevalence of 35.2%, an LDAR sensitivity of 86.7%, a specificity of 96.7%, and an absolute precision of 5%, the minimum required total sample size was approximately 504 patients. The required number of AKI cases for sensitivity estimation was 178, and the required number of non-AKI cases for specificity estimation was 50. The present cohort included 512 patients, including 180 with AKI and 332 without AKI, and therefore met these requirements.For multivariable logistic regression, sample size adequacy was additionally assessed using the events-per-variable approach. With 180 AKI events and four predictors retained in the final model, the model had 45 events per variable, which was considered sufficient to limit overfitting.

Missing data were assessed for all study variables and summarized as counts and percentages. Because missingness was below 5% for variables included in the primary analyses, a complete-case analysis was used, and multiple imputation was not performed.

## Results

A total of 512 patients were included, of whom 180 (35.2%) were classified as having creatinine-defined AKI according to KDIGO serum creatinine criteria. The median age was 43 years (IQR 31–57), and 374 (73.0%) were male. Age and gender did not differ between the AKI and non-AKI groups (*p* = 0.138 and *p* = 0.253, respectively). Earthquake-related cases accounted for 307 (60.0%) of presentations. Hemodialysis was required during hospitalization in 78 (43.3%) patients with AKI. Injury severity, measured by the Injury Severity Score (ISS), was higher in the AKI group (14 [IQR 9–17] vs. 9 [IQR 8–13]; *p* < 0.001). Overall, 29 (5.7%) patients died during hospitalization; mortality was higher among those with AKI (15.0% vs. 0.6%; *p* < 0.001). Baseline characteristics and clinical course variables are summarized in Table 1.

**Table 1:** Baseline characteristics and clinical course of patients with crush syndrome by acute kidney injury (AKI) status

	TOTAL (*n* = 512)		AKI NOT PRESENT (*n* = 332)	AKI PRESENT (*n* = 180)	*p* value
**Age** Median (IQR)	43 (31–57)	42 (31.25-53)		45 (29–60)	0.138*
**Gender**					
Male	374 (73.0)	248 (74.7)		126 (70.0)	0.253
Female	138 (27.0)	84 (25.3)		54 (30.0)	
**Etiology**					
Other trauma	205 (40.0)	126 (38.0)		79 (43.9)	0.191
Earthquake	307 (60.0)	206 (62.0)		101 (56.1)	
**Comorbidity**					
DM	80 (15.6)	25 (7.5)		55 (30.6)	< 0.001**
HT	62 (12.1)	37 (11.1)		25 (13.9)	0.363
CAD	50 (9.8)	27 (8.1)		23 (12.8)	0.091
Other	63 (12.3)	35 (10.5)		28 (15.6)	0.099
**Site of Injury**					
Upper extremity	113 (22.1)	91 (27.4)		22 (12.2)	< 0.001**
Lower extremity	149 (29.1)	77 (23.2)		72 (40.0)	
Abdomen	250 (48.8)	164 (49.4)		86 (47.8)	
**Blood Transfusion**					
No	433 (84.6)	302 (91.0)		131 (72.8)	< 0.001**
RBC	79 (15.4)	30 (9.0)		49 (27.2)	
**Vasopressor Need**					
No	343 (67.0)	218 (65.7)		125 (69.4)	0.385
Yes	169 (33.0)	114 (34.3)		55 (30.6)	
**Hemodialysis Need**					
No	434 (84.8)	332 (100.0)		102 (56.7)	< 0.001**
Yes	78 (15.2)	0 (0.0)		78 (43.3)	
**Amputation**					
Performed	60 (11.7)	0 (0.0)		60 (33.3)	< 0.001**
Not performed	452 (88.3)	332 (100.0)		120 (66.7)	
**Mortality**					
Died	29 (5.7)	2 (0.6)		27 (15.0)	< 0.001**
Surviving	483 (94.3)	330 (99.4)		151 (85.0)	
**Injury Severity Score** Median (IQR)	9 (9–16)	9 (8–13)		14 (9–17)	< 0.001*
**Duration of Hospitalization** Median (IQR)	6.50 (3.00–17.00)	5.00 (3.00–10.00)		13.00 (5.00-38.75)	< 0.001*

AKI: Acute kidney injury; IQR: Interquartile range; DM: Diabetes mellitus; HT: Hypertension; CAD: Coronary artery disease; RBC: Red blood cell

*Mann-Whitney U test; ** Ki- square test; *p* < 0,05 statistically significant

Admission laboratory parameters are shown in Table 2. Compared with patients without AKI, those with AKI had higher white blood cell and neutrophil counts and lower hemoglobin and platelet levels (*p* < 0.001 for each). Markers of tissue injury were also higher in the AKI group, including LDH and CK (*p* < 0.001 for both). Albumin concentrations were lower in the AKI group, and the LDAR was higher (*p* < 0.001 for both).

**Table 2:** Admission clinical and laboratory parameters in patients with crush syndrome by acute kidney injury (AKI) status

	TOTAL (*n* = 512)	AKI NOT PRESENT (*n* = 332)	AKI PRESENT (*n* = 180)	*p* value
**WBC** Median (IQR)	12.29 (9.63–16.35)	11.49 (8.96–14.82)	14.44 (10.49–19.04)	< 0.001*
**Hgb** Median (IQR)	13.50 (11.42-15.00)	13.90 (12.10-15.07)	12.50 (10.12–14.67)	< 0.001*
**Plt** Median (IQR)	230.50 (170.25–279.00)	237.50 (202.00-287.00)	205.50 (142.25–256.00)	< 0.001*
**Neutrophil** Median (IQR)	9.23 (6.46–13.05)	8.66 (6.30–11.40)	10.88 (7.13–15.53)	< 0.001*
**Lymphocyte** Median (IQR)	1.63 (1.13–2.37)	1.69 (1.15–2.36)	1.55 (1.08–2.52)	0.357
**Creatinine** Median (IQR)	0.71 (0.58–2.30)	0.62 (0.45–0.73)	2.54 (2.28–3.83)	< 0.001*
**Urea** Median (IQR)	39 (28.25-79.00)	31 (25–40)	90.50 (76.00–99.00)	< 0.001*
**LDH** Median (IQR)	705.50 (384.00-995.50)	408.00 (364.00-711.00)	1307.00 (844.00-1462.25)	< 0.001*
**Albumin** Median (IQR)	40.60 (33.62–44.50)	42.40 (36.92–45.27)	34.70 (27.82–41.47)	< 0.001*
**CK** Median (IQR)	1974.00 (1716.75-41036.50)	1802.50 (1134.75–1991.00)	53092.50 (38753.50-66794.75)	< 0.001*
**ALT** Median (IQR)	111 (77–179)	86 (65–113)	249.50 (164.50-351.25)	< 0.001*
**AST** Median (IQR)	253.50 (175.25–581.50)	189 (140–255)	839.00 (543.75-1083.75)	< 0.001*
**Na** Median (IQR)	138 (136–140)	139.00 (137.25–141.00)	136 (133–138)	< 0.001*
**K** Median (IQR)	4.11 (3.83–4.48)	4.11 (3.87–4.41)	4.12 (3.69–4.61)	0.874
**pH** Median (IQR)	7.40 (7.33–7.41)	7.40 (7.40–7.42)	7.28 (7.15–7.34)	< 0.001*
**PCO₂** Median (IQR)	33 (31-37.40)	32.40 (31.00-36.27)	33.50 (31.00-38.77)	0.120
**HCO**^**3**^^−^ Median (IQR)	18.70 (17.60–20.00)	19.10 (18.20–21.20)	18.00 (14.10–18.80)	< 0.001*
**Lactate** Median (IQR)	1.55 (1.46–1.87)	1.54 (1.46–1.63)	1.89 (1.48–2.22)	< 0.001*
**Base deficit** Median (IQR)	-7.30 [(-8.10)-(-6.70)]	-7.30 [(-8.20)-(-6.70)]	-7.30 [(-8.10)-(-6.80)]	< 0.001*
**LDAR** Median (IQR)	17.15 (9.59–29.22)	11.70 (8.48–17.23)	33.72 (27.61–43.60)	< 0.001*

AKI: Acute kidney injury; IQR: Interquartile range; WBC: White blood cell; Hgb: Hemoglobin; Plt: Platelet; LDH: Lactate dehydrogenase; CK: Creatine kinase; ALT: Alanine transaminase; AST: Aspartate aminotransferase; LDAR: Lactate dehydrogenase-to-albumin ratio

*Mann-Whitney U test, *p* < 0,05 statistically significant

In univariable analyses of admission and clinically relevant pre-outcome variables, multiple parameters were associated with AKI. Length of hospitalization was not included in the predictive analyses because it is a post-admission clinical course variable. In the final multivariable logistic regression model, diabetes mellitus, ALT, LDAR, and bicarbonate remained independently associated with AKI after adjustment. In addition, LDAR was identified as an independent predictor of RRT requirement (*p* < 0.05) (Table 3).

**Table 3:** Univariable and multivariable logistic regression analyses of factors associated with acute kidney injury and renal replacement therapy requirement in patients with crush syndrome

	Univariable		Multivariable		
	OR (95%CI)	*p* value	OR (95%CI)		*p* value
**Prediction of AKI**					
DM	5.403 (3.224–9.056)	< 0.001	10.887 (2.500-47.414)		< 0.001
Urea	1.088 (1.074–1.103)	< 0.001			
LDH	1.008 (1.006–1.009)	< 0.001			
CK	1.002 (1.000-1.186)	< 0.001			
ALT	1.085 (1.062–1.109)	< 0.001	1.098 (1.051–1.148)		< 0.001
LDAR	1.400 (1.313–1.493)	< 0.001	1.277 (1.161–1.405)		< 0.001
Albumin	0.894 (0.870–0.918)	< 0.001			
HCO₃⁻	0.652 (0.594–0.716)	< 0.001	0.558 (0.419–0.743)		< 0.001
**Overall model fit**			χ^2^ = 594.930 *p* < 0.001 Nagelkerke R²=0.946		
**Hosmer–Lemeshow test**			χ2 = 1.692 *p* = 0.989		
**Prediction of RRT requirement**					
Injury Severity Score	1.086 (1.048–1.125)	< 0.001			
Creatinine	3.196 (2.536–4.026)	< 0.001	1.780 (1.315–2.411)		< 0.001
Urea	1.049 (1.038–1.059)	< 0.001			
LDH	1.004 (1.003–1.005)	< 0.001			
CK	1.000 (1.000–1.000)	< 0.001	1.000 (1.000–1.000)		< 0.001
AST	1.004 (1.003–1.005)	< 0.001			
LDAR	1.088 (1.067–1.110)	< 0.001	1.035 (1.015–1.056)		< 0.001
Albumin	0.919 (0.892–0.947)	< 0.001			
HCO₃⁻	0.838 (0.775–0.906)	< 0.001			
Overall model fit			χ^2^ = 193.013 *p* < 0.001 Nagelkerke R²=0.547		
Hosmer–Lemeshow test			**χ2 = 9.439 *p* = 0.307		

AKI: Acute kidney injury; DM: Diabetes mellitus; LDH: Lactate dehydrogenase; CK: Creatine kinase; ALT: Alanine transaminase; LDAR: Lactate dehydrogenase-to-albumin ratio; AST: Aspartate aminotransferase; OR: Odds ratio; CI: Confidence interval; RRT: Renal replacement therapy

*p* < 0.05 was considered statistically significant. For diabetes mellitus, the reference category was the absence of diabetes

The Hosmer–Lemeshow test was used to assess model fit

**Fig. 2:**
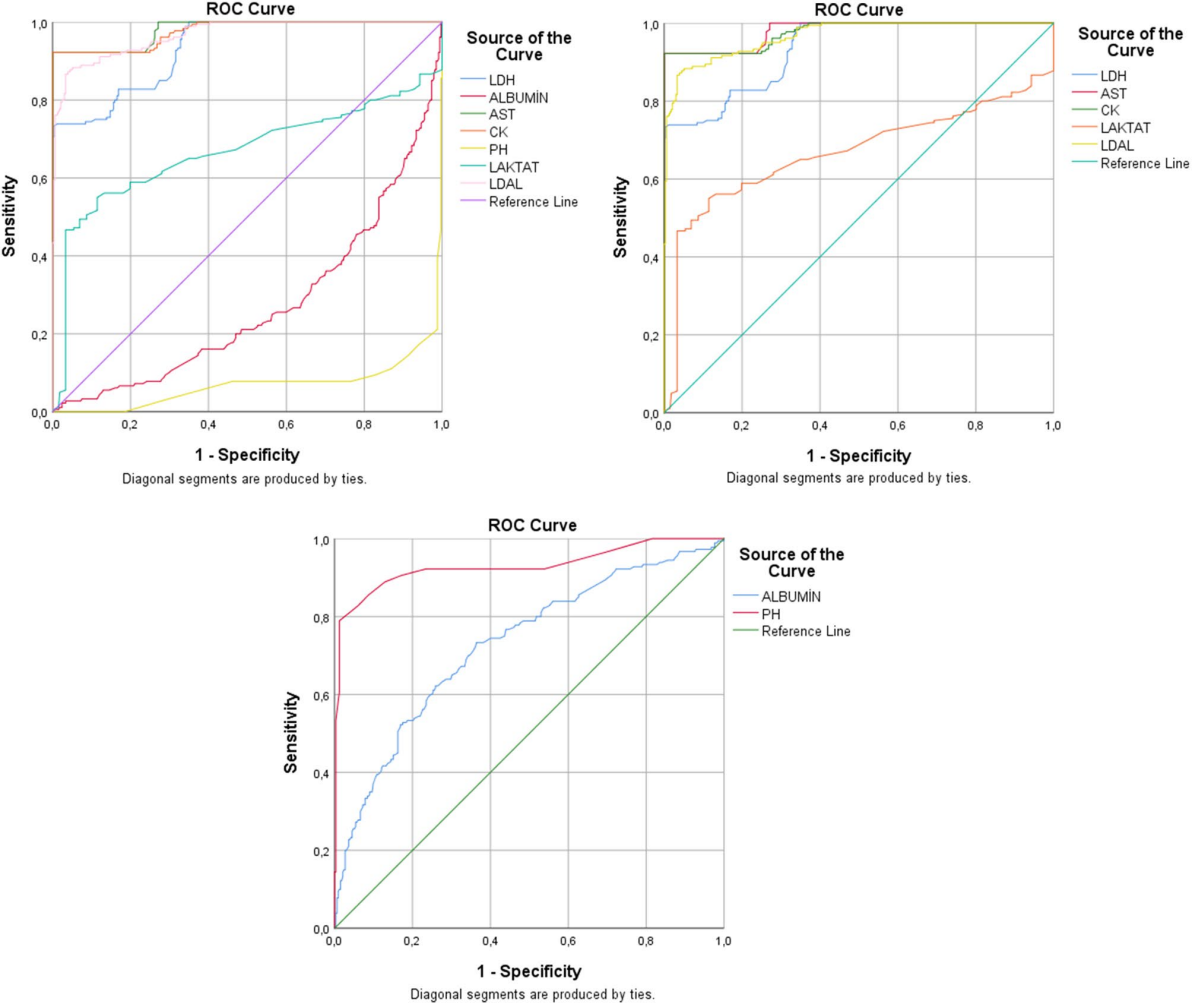
ROC analysis diagrams

ROC analyses of admission variables are presented in Table 4; Fig. 2. CK showed the highest discrimination for AKI (AUC 0.977, 95% CI 0.964–0.989; *p* < 0.001), followed by LDAR (AUC 0.966, 95% CI 0.953–0.980; *p* < 0.001). Using a Youden-derived LDAR cut-off of ≥ 23.59, sensitivity was 86.7%, and specificity was 96.7% (Youden index = 0.834). Additional AUCs are reported in Table 4, including LDH 0.932, pH 0.893, and albumin 0.779.

**Table 4:** Receiver operating characteristic (ROC) analysis of admission clinical and laboratory variables for discrimination of acute kidney injury (AKI)

Variables	AUC	*p* value	95%CI	Cut-off	Sensitivity (%)	Specificity (%)	Youden Index	PPV (%)	NPV (%)
Injury Severity Score	0.715	< 0.001*	0.668–0.762	10.50	65.0	69.9	0.349	53.9	78.6
Neutrophil	0.615	< 0.001*	0.562–0.667	9.98	57.2	64.2	0.214	46.3	73.4
LDH	0.932	< 0.001*	0.912–0.953	614.00	98.9	66.0	0.649	61.1	99.0
CK	0.977	< 0.001*	0.964–0.989	10160.0	92.2	99.7	0.919	99.4	95.9
AST	0.905	< 0.001*	0.889–0.921	334.50	82.2	93.4	0.816	88.8	92.9
pH	0.893	< 0.001*	0.852–0.928	7.34	78.9	98.8	0.777	97.0	86.7
LDAR	0.966	< 0.001*	0.953–0.980	23.59	86.7	96.7	0.834	93.4	93.0
Albumin	0.779	< 0.001*	0.732–0.8705	40.25	78.3	66.6	0.569	52.1	81.4
HCO₃⁻	0.754	< 0.001*	0.711–0.798	18.05	50.6	85.2	0.358	65.0	76.0
Lactate	0.667	< 0.001*	0.609–0.726	1.84	55.0	88.6	0.436	72.2	78.4

LDH: Lactate dehydrogenase; CK: Creatine kinase; AST: Aspartate aminotransferase; LDAR: Lactate dehydrogenase-to-albumin ratio; AUC: Area under the curve; CI: Confidence interval; PPV: Positive predictive value; NPV: Negative predictive value. * *p* < 0,05 statistically significant

In the DeLong comparison of diagnostic performance for AKI, the AUC difference between LDAR and CK was 0.010 and was not statistically significant (*p* = 0.124; 95% CI: −0.003 to 0.023). These findings suggest that LDAR demonstrated diagnostic performance comparable to that of CK in this cohort, although external validation is required (Table 5).

**Table 5:** DeLong comparison of receiver operating characteristic (ROC) curves for acute kidney injury (AKI) discrimination

Variable	AUC	*p* value	95%CI
LDH	0.932	< 0.001*	0.912–0.953
LDAR	0.966	< 0.001*	0.953–0.980
Albumin	0.729	< 0.001*	0.682–0.775
CK	0.977	< 0.001*	0.964–0.989
LDAR vs. LDH	0.034	< 0.001*	0.019–0.049
LDAR vs. Albumin	0.238	< 0.001*	0.194–0.282
LDH vs. Albumin	0.204	< 0.001*	0.151–0.257
LDAR vs. CK	0.010	0.124	-0.003-0.023
LDH vs. CK	0.044	< 0.001*	0.025–0.063
Albumin vs. CK	0.248	< 0.001*	0.202–0.295

AKI: Acute kidney injury; ROC: Receiver operating characteristic; AUC: Area under the curve; CI: Confidence interval; LDH: Lactate dehydrogenase; LDAR: Lactate dehydrogenase-to-albumin ratio; CK: Creatine kinase

* *p* < 0.05 statistically significant

Similar to its performance for AKI discrimination, LDAR was identified as a strong composite biomarker for discriminating RRT requirement in patients with crush syndrome (AUC 0.905; 95% CI 0.878–0.932). An LDAR cut-off of ≥ 22.68 provided 97.4% sensitivity and 77.6% specificity, with a Youden index of 0.751 (Table 6).

**Table 6:** Receiver operating characteristic (ROC) analysis of clinical and laboratory parameters for discrimination of renal replacement therapy (RRT) requirement in patients with crush syndrome

Variable	AUC	*p* value	95% CI	Cut-off	Sensitivity (%)	Specificity (%)	Youden Index	PPV (%)	NPV (%)
Injury Severity Score	0.640	< 0.001*	0.569–0.711	12.50	59.0	64.3	0.233	22.8	89.7
Neutrophil count	0.652	< 0.001*	0.583–0.721	10.51	62.8	64.1	0.269	23.7	90.5
Creatinine	0.909	< 0.001*	0.884–0.934	2.12	98.7	79.7	0.784	46.9	99.0
Urea	0.893	< 0.001*	0.865–0.921	51.50	98.7	72.8	0.715	39.4	99.6
LDH	0.890	< 0.001*	0.856–0.924	949.50	83.3	84.8	0.681	49.6	96.5
CK	0.889	< 0.001*	0.861–0.916	31512.50	98.7	80.2	0.789	47.2	99.7
AST	0.898	< 0.001*	0.872–0.924	414.50	98.7	79.7	0.784	46.6	99.7
LDAR	0.905	< 0.001*	0.878–0.932	22.68	97.4	77.6	0.751	43.9	99.4
Albumin	0.692	< 0.001*	0.629–0.755	34.15	48.7	77.4	0.261	27.9	89.3
HCO₃⁻	0.640	< 0.001*	0.576–0.705	18.75	69.2	50.9	0.202	20.2	90.2
pH	0.823	< 0.001*	0.774–0.872	7.34	78.2	80.4	0.586	40.2	93.6

AUC: Area under the curve; CI: Confidence interval; PPV: Positive predictive value; NPV: Negative predictive value; LDH: Lactate dehydrogenase; CK: Creatine kinase; AST: Aspartate aminotransferase; LDAR: Lactate dehydrogenase-to-albumin ratio

* *p* < 0.05 statistically significant

## Discussion

Crush syndrome (CS) can follow both mass-casualty events and high-energy trauma and remains clinically important because AKI and related metabolic complications drive much of the associated morbidity and mortality [2, 3]. In the emergency department, early recognition of patients requiring RRT and high risk of AKI is essential to guide timely resuscitation, electrolyte management, and, when required, escalation of care [2–16]. In our cohort, CK showed the highest discriminatory performance for AKI, confirming its central role in the assessment of rhabdomyolysis in patients with CS. However, CK levels can be influenced by factors such as muscle mass and the timing of sampling relative to injury, which may limit its ability to consistently support early risk stratification across patients [16, 17]. Therefore, LDAR should not be interpreted as a replacement for CK, but rather as a complementary admission-based index that combines tissue injury, reflected by LDH, with albumin-related systemic inflammatory burden and physiological reserve. LDAR was markedly higher in patients classified as having AKI than in those who did not (33.72 vs. 11.70; *p* < 0.001) and demonstrated high discrimination for AKI (AUC 0.966), although its AUC was slightly lower than that of CK (AUC 0.977). Because the exact timing of AKI onset relative to ED presentation could not be reliably determined in all patients, this finding should be interpreted as admission-based discrimination of creatinine-defined AKI rather than as pure prediction of new-onset AKI after admission. In univariable analyses, several admission parameters were associated with AKI, whereas diabetes mellitus, alanine aminotransferase, LDAR, and bicarbonate remained independently associated with AKI after multivariable adjustment. Furthermore, beyond its performance in discriminating AKI, LDAR also showed high discrimination for RRT requirement, supporting its potential role as a composite admission-based marker of severe renal involvement in CS (AUC 0.905; 95% CI 0.878–0.932).

In their study following the Marmara earthquake, Sever et al. reported that 12% of patients treated in the hospital developed renal problems and 8.9% required renal replacement therapy (RRT) [18]. In a study by Comoğlu et al., conducted after the 6 February 2023 Kahramanmaraş earthquake, 314 patients with CK > 1,000 U/L were included; 30.2% patients were classified as having AKI, and 21.6% required RRT [19]. Additionally, Long et al. reported that mortality rates approach 20% in CS patients who develop AKI and increase further in those who develop multiple organ failure [2]. Consistent with the literature, 35.2% (*n* = 180) of 512 patients in our cohort were classified as having AKI, 43.3% (*n* = 78) required RRT, and 15.0% of patients with AKI (27/180) died.

Trauma severity and the extent of muscle involvement are key determinants of AKI in crush syndrome [1]. In our study, patients classified as having AKI had higher injury severity (ISS 14 vs. 9; *p* < 0.001). In their study of pediatric earthquake victims, Atmis et al. found that the percentage of crushed body surface area strongly discriminated dialysis need (AUC 0.907), with an optimal cutoff of 30%; they also reported that each 10% increase in crushed body surface area markedly increased dialysis requirement [20]. Comoglu et al. reported that the number of traumatized sites is an independent risk factor for RRT (OR = 2.2) [19]. In our study, we categorized injury location as lower extremity, upper extremity, and other areas and observed a significant association between injury site and AKI development (*p* < 0.001). The higher incidence of AKI with lower-extremity and abdominal involvement may be related to greater skeletal muscle mass in these regions and, consequently, a larger myoglobin load.

The fundamental pathophysiological mechanisms contributing to AKI in crush syndrome include renal hypoperfusion, myoglobin-associated tubular obstruction, and direct tubular injury [21]. Comoglu et al. reported that CK effectively discriminated AKI (AUC 0.928) and proposed an optimal CK cutoff of 23,000 U/L [19]. Similarly, Atmis et al. demonstrated a positive correlation between serum creatinine and CK in pediatric earthquake victims. They reported that CK levels above 40,000 U/L predicted the need for dialysis with 86% sensitivity and 83% specificity [20]. We also found that CK had the highest discriminatory performance for AKI in our cohort (AUC 0.977), with an optimal cutoff of 10,160 U/L. Although this threshold was lower than that reported in some previous series, we observed high diagnostic accuracy (92.2% sensitivity and 99.7% specificity), which may reflect differences in case mix and the timing of ED presentation and sampling.

LDH is an intracellular enzyme present in most tissues and is a recognized marker of cellular injury. In cells entering a hypoxic–ischemic state, LDH levels can rise within minutes [22, 23]. Legrand et al. demonstrated in culture that LDH activity reliably reflects the number of dead cells [24]. For these reasons, LDH has been linked to adverse trajectories and 28-day mortality in conditions such as sepsis, ischemic stroke, trauma, and acute renal failure [23, 25]. In critically ill patients with AKI, higher LDH has also been reported as an independent predictor of in-hospital mortality, and incorporation of LDH into severity scoring can improve discriminatory performance [26]. In hypoxic and ischemic processes following trauma, activation of glycolysis is thought to contribute to increased LDH levels [23]. In our cohort, LDH showed strong discriminatory performance in ROC analysis (AUC 0.932) and was closely aligned with AKI risk, supporting the concept that multi-tissue injury in crush syndrome elevates LDH and that LDH may reflect overall tissue injury burden in CS and its association with AKI.

Albumin is widely used to assess nutritional status and inflammatory processes [27]. Following crush injuries, plasma leakage from wounds, an increased catabolic state, malnutrition, fluid overload, and infectious complications can contribute to hypoalbuminemia [28]. Hypoalbuminemia has been associated with worse outcomes in AKI [16]. In the study by Comoglu et al., albumin was reported to predict both AKI and RRT [16]. Hu et al. reported albumin as a predictor of AKI in patients with crush injuries, with a cutoff value of 2.7 mg/dL [29]. Consistent with these reports, serum albumin levels were significantly lower in patients with AKI in our cohort (34.70 vs. 42.40 g/L; *p* < 0.001), and the AUC of albumin for AKI discrimination was 0.779.

By integrating organ failure, chronic diseases, inflammation, and nutritional status, LDAR may provide a more comprehensive prognostic signal than LDH or albumin alone by reflecting tissue injury alongside physiological reserve. In a retrospective cohort of critically ill patients with acute respiratory distress syndrome, Zhang et al. reported that higher LDAR values were associated with 30-day, 90-day, and in-hospital mortality [30]. Similarly, in a sepsis cohort, Guan et al. identified a nonlinear association between LDAR and all-cause ICU mortality and suggested a cutoff of ≥ 10.57 as an independent risk factor for death [31]. In critically ill patients with AKI, Deng et al. reported that the higher LDAR group had significantly higher in-hospital mortality across AKI stages 1–3 [32]. In our ROC analysis, the AUC for LDAR was 0.966 (95% CI 0.953–0.980), and a cutoff of ≥ 23.59 yielded 86.7% sensitivity and 96.7% specificity. These findings suggest that LDAR is a strong admission biomarker for early discrimination of AKI risk in CS patients. In addition, we showed that LDAR had a high AUC for discriminating between patients with and without RRT requirements in patients with CS, with a cut-off of ≥ 22.68 yielding 97.4% sensitivity and 77.6% specificity.

However, we believe that an LDAR above this threshold alone warrants more rigorous renal monitoring, including repeated creatinine and electrolyte measurements, close monitoring of acid-base balance, strict monitoring of urine output when possible, and careful, individualized fluid replacement based on hemodynamic status and the risk of volume overload. Additionally, in patients with concurrent elevated CK, rising creatinine, hyperkalemia, metabolic acidosis, oliguria, or hemodynamic instability, a high LDAR may support earlier nephrology consultation or intervention.

This study has several strengths, including a large cohort (*n* = 512), representation of both earthquake-related and non-earthquake trauma cases, and a comprehensive assessment of routinely available admission laboratory parameters. Using both ROC analysis and multivariable regression, we provide clinically interpretable estimates supporting the potential utility of LDAR at ED presentation for AKI risk stratification and RRT. Nonetheless, the findings should be interpreted in light of important limitations. The retrospective, single-center design is a significant limitation and warrants careful consideration of external validity. Although the cohort included both earthquake-related and non-earthquake-related crush syndrome cases, the case mix was shaped by regional referral patterns, the context of the 6 February 2023 earthquake, local trauma care pathways, prehospital transfer conditions, surgical decision-making, and institutional dialysis capacity. Therefore, the diagnostic performance and the Youden-derived LDAR threshold observed in this study may not be directly generalizable to other hospitals, regions, disaster settings, or trauma systems. In particular, the relative proportions of earthquake-related entrapment injuries and non-earthquake high-energy trauma may influence biomarker distributions, AKI incidence, and the need for RRT. For this reason, the LDAR cut-off should be considered exploratory and hypothesis-generating rather than a universally applicable clinical threshold. Prospective multicenter external validation studies are needed to confirm LDAR’s performance across different crush syndrome populations, care settings, and etiological subgroups. Residual confounding related to treatment heterogeneity is another important limitation. AKI development in crush syndrome is strongly influenced by early resuscitation quality, time from injury to admission, duration of compression or entrapment, hemodynamic instability, urine alkalinisation, mannitol use, and surgical/perioperative factors. In this retrospective cohort, detailed prehospital fluid volume, post-admission fluid rate and cumulative volume, timing and duration of urine alkalinisation, mannitol dose and timing, compression duration, and duration of pre-/intraoperative hypotension were not consistently documented. Therefore, these variables could not be reliably included in the multivariable models. As a result, residual confounding and confounding by indication remain possible, and the observed association between LDAR and AKI should not be interpreted as causal. From a pragmatic standpoint, because LDH and albumin are widely available on admission, LDAR may offer a simple adjunct to early clinical assessment to help identify patients who warrant closer monitoring and timely escalation of care. Another important limitation relates to AKI ascertainment. Urine output criteria were not included in the primary AKI definition because hourly urine output was not consistently documented in the retrospective emergency department records.

The very high AUC values observed for CK and LDAR should also be interpreted cautiously. These estimates may partly reflect spectrum effects associated with a selected crush syndrome cohort, the contrast between clearly severe and clearly non-AKI cases, single-center referral patterns, and creatinine-defined outcome ascertainment. Therefore, the apparent discriminatory performance may be optimistic and should not be assumed to generalize to broader emergency or disaster populations without external validation.

## Conclusion

In this retrospective cohort of patients with crush syndrome, admission LDAR demonstrated high discrimination for creatinine-defined acute kidney injury and remained independently associated with AKI after adjustment. Because AKI and RRT onset relative to emergency department presentation could not be reliably determined in all patients, LDAR should be interpreted as an admission-based early identification and risk stratification marker rather than as a stand-alone predictor of new-onset AKI. As LDH and albumin are routinely measured in the emergency department, LDAR can be calculated without additional testing and may complement, but not replace, CK and clinical assessment in early renal risk stratification, including in mass-casualty contexts. Prospective multicenter external validation is required before the LDAR threshold identified in this single-center cohort can be applied as a clinical decision threshold in other trauma systems, disaster settings, or etiological subgroups of crush syndrome.

## Data Availability

The datasets used and analysed during the current study are not publicly available due to restrictions related to patient privacy and institutional data governance but are available from the corresponding author on reasonable request.

## Abbreviations

AKI: Acute kidney injury
ALT: Alanine aminotransferase
AST: Aspartate aminotransferase
AUC: Area under the curve
CI: Confidence interval
CK: Creatine kinase
CS: Crush syndrome
ED: Emergency department
HCO₃⁻: Bicarbonate
ICU: Intensive care unit
ISS: INJURY Severity Score
KDIGO: Kidney Disease: Improving Global Outcomes
LDAR: Lactate dehydrogenase-to-albumin ratio
LDH: Lactate dehydrogenase
NPV: Negative predictive value
OR: Odds ratio
PCO₂: Partial pressure of carbon dioxide
PPV: Positive predictive value
RBC: Red blood cell
ROC: Receiver operating characteristic
RRT: Renal replacement therapy
WBC: White blood cell
